# Outcomes from an inpatient beta-lactam allergy guideline across a large US health system

**DOI:** 10.1017/ice.2019.50

**Published:** 2019-05

**Authors:** Kimberly G. Blumenthal, Yu Li, Joyce T. Hsu, Anna R. Wolfson, David N. Berkowitz, Victoria A. Carballo, Jesse M. Schwartz, Kathleen A. Marquis, Ramy Elshaboury, Ronak G. Gandhi, Barbara B. Lambl, Monique M. Freeley, Alana Gruszecki, Paige G. Wickner, Erica S. Shenoy

**Affiliations:** 1 Division of Rheumatology, Allergy, and Immunology, Department of Medicine, Massachusetts General Hospital, Boston, Massachusetts; 2 The Mongan Institute, Massachusetts General Hospital, Boston, Massachusetts; 3 Harvard Medical School, Boston, Massachusetts; 4 University of Pittsburgh School of Medicine, Pittsburgh, Pennsylvania; 5 Division of Rheumatology, Allergy and Immunology, Department of Medicine, Brigham and Women’s Hospital, Boston, Massachusetts; 6 Department of Pharmacy, Newton-Wellesley Hospital, Newton, Massachusetts; 7 Partners HealthCare System, Quality, Safety, and Value, Boston, Massachusetts; 8 Division of Allergy and Clinical Immunology, Jewish General Hospital, McGill University, Montreal, Quebec, Canada; 9 Department of Pharmacy, Brigham and Women’s Hospital, Boston, Massachusetts; 10 Department of Pharmacy, Massachusetts General Hospital, Boston, Massachusetts; 11 Division of Infectious Diseases, Department of Medicine, North Shore Medical Center, Salem, Massachusetts; 12 Pharmacy Department, North Shore Medical Center, Salem, Massachusetts; 13 Pharmacy Department, Brigham and Women’s Faulkner Hospital, Boston, Massachusetts; 14 Division of Infectious Diseases, Department of Medicine, Massachusetts General Hospital, Boston, Massachusetts; 15 Infection Control Unit, Massachusetts General Hospital, Boston, Massachusetts

## Abstract

**Objective:**

To assess the safety of, and subsequent allergy documentation associated with, an antimicrobial stewardship intervention consisting of test-dose challenge procedures prompted by an electronic guideline for hospitalized patients with reported β-lactam allergies.

**Design:**

Retrospective cohort study.

**Setting:**

Large healthcare system consisting of 2 academic and 3 community acute-care hospitals between April 2016 and December 2017.

**Methods:**

We evaluated β-lactam antibiotic test-dose outcomes, including adverse drug reactions (ADRs), hypersensitivity reactions (HSRs), and electronic health record (EHR) allergy record updates. HSR predictors were examined using a multivariable logistic regression model. Modification of the EHR allergy record after test doses considered relevant allergy entries added, deleted, and/or specified.

**Results:**

We identified 1,046 test-doses: 809 (77%) to cephalosporins, 148 (14%) to penicillins, and 89 (9%) to carbapenems. Overall, 78 patients (7.5%; 95% confidence interval [CI], 5.9%–9.2%) had signs or symptoms of an ADR, and 40 (3.8%; 95% CI, 2.8%–5.2%) had confirmed HSRs. Most HSRs occurred at the second (ie, full-dose) step (68%) and required no treatment beyond drug discontinuation (58%); 3 HSR patients were treated with intramuscular epinephrine. Reported cephalosporin allergy history was associated with an increased odds of HSR (odds ratio [OR], 2.96; 95% CI, 1.34–6.58). Allergies were updated for 474 patients (45%), with records specified (82%), deleted (16%), and added (8%).

**Conclusion:**

This antimicrobial stewardship intervention using β-lactam test-dose procedures was safe. Overall, 3.8% of patients with β-lactam allergy histories had an HSR; cephalosporin allergy histories conferred a 3-fold increased risk. Encouraging EHR documentation might improve this safe, effective, and practical acute-care antibiotic stewardship tool.

Beta-lactam antibiotic allergies, reported by up to 15% of hospitalized patients, impact acute-care antibiotic prescribing.^1^^,^^2^ Cephalosporins, antibiotics important to the treatment of common inpatient infections, are inconsistently prescribed to patients reporting penicillin allergies despite low cross reactivity.^3^^–^^5^ Alternatives to β-lactam antibiotics may be less effective^4^^,^^6^ and can lead to adverse sequelae for patients, most notably healthcare-associated infections such as *Clostridioides difficile* infection.^7^^,^^8^

Most patients with a documented penicillin allergy do not have clinically significant hypersensitivity and can be safely treated with penicillins and other β-lactams.^9^ Although penicillin allergy evaluation is encouraged by antibiotic stewardship guidelines,^10^ penicillin skin testing (PST) is operationally challenging in acute-care settings.^11^^,^^12^ Furthermore, many of the antibiotics generally used in hospitalized patients after a negative PST can be administered safely without preceding PST with a full-dose or test-dose challenge.^13^^–^^15^

The Partners HealthCare System (PHS) guideline for inpatients with β-lactam allergy histories is an antibiotic stewardship tool that includes penicillin and cephalosporin hypersensitivity pathways that direct PST when institutionally available and needed (ie, patients reporting IgE-mediated allergy symptoms to a penicillin who require a penicillin or cross-reactive cephalosporin), but it encourages direct full-dose and test-dose (ie, standardized 2-step graded) drug challenges. Prior to this study, our results demonstrated that the guideline safely increased β-lactam antibiotic use at 2 academic medical centers.^12^^,^^14^^–^^16^ In this study, we sought to further assess the safety of guideline-directed β-lactam antibiotic test doses after implementation of the computerized guideline throughout 5 acute-care PHS hospitals with varied resources.

## Methods

### Computerized guideline with optional clinical decision support

We developed β-lactam hypersensitivity pathways in 2013 at the Massachusetts General Hospital (MGH), which were modified and studied prospectively at the Brigham and Women’s Hospital (BWH).^12^^,^^14^^,^^16^ All pathways were implemented as hospital guidelines with electronic health record (EHR) support throughout PHS acute-care sites in 2016 (Supplemental Table 1 online).^15^

**Table 1.** Test Dose and Patient Characteristics

Variable	No. (%) (n = 1,046)
***Test-dose characteristics*** ^**a**^	
**β-lactam drug class**	
Penicillin	148 (14)
Cephalosporin	809 (77)
Carbapenem	89 (9)
**Hospital type**	
Academic^b^	867 (83)
Community^c^	179 (17)
**Ordering provider**	
House staff	621 (59)
Physician assistant	144 (14)
Attending physician	142 (14)
Nurse practitioner	120 (12)
Unknown	19 (2)
**Service**	
Internal medicine^d^	469 (45)
Emergency department	131 (13)
Surgery^d^	126 (12)
Oncology	111 (11)
Intensive care	110 (11)
Cardiology^d^	24 (2)
Neurology^d^	16 (2)
Obstetrics/Gynecology^d^	13 (1)
Pediatrics^d^	11 (1)
Unknown	35 (3)
Allergy/Immunology consultation	96 (9)
Penicillin skin test performed	38 (4)
Days in hospital prior to test dose, median (IQR)	2 [1, 4]
Length of stay, median (IQR)	10 [5, 19]
***Patient Characteristics*** ^**a**^	**n = 942**
Female	612 (65)
Age at admission, median y (IQR)	64 [51, 75]
Allergy to penicillin	900 (96)
**Penicillin reaction** ^**e**^	
Nonsevere cutaneous^f^	453 (48)
Severe IgE^g^	185 (20)
Allergy to cephalosporin	273 (29)
**Cephalosporin reaction** ^**h**^	
Nonsevere cutaneous^i^	164 (17)
Severe IgE^j^	42 (5)
**Other drug allergy**	
Sulfonamide antibiotics	254 (27)
Opioids	170 (18)
Fluoroquinolones	128 (14)
Macrolides	106 (11)

Note. IQR, interquartile range; Ig, immunoglobulin; MGH, Massachusetts General Hospital; BWH, Brigham and Women’s Hospital; NWH, Newton Wellesley Hospital; NSMC, North Shore Medical Center; BWF, Brigham and Women’s Faulkner Hospital.

a Number (%) unless otherwise specified.

b MGH performed 713 and BWH performed 154.

c NWH performed 89, NSMC performed 80, and BWF performed 10.

d Non-intensive care.

e Numbers do not sum because patients can have >1 reaction. Reactions also included 214 other reactions and 197 with unknown reactions.

f Nonsevere cutaneous reactions to penicillin included rash (n = 266), hives (n = 190), itching (n = 35), and flushing (n = 2).

g Severe IgE reactions to penicillin included anaphylaxis (n = 78), angioedema (n = 48), swelling (n = 40), shortness of breath (n = 21), bronchospasm (n = 6), wheezing (n = 6), syncope (n = 5), dizziness (n = 3), and tested positive (n = 1).

h Numbers do not sum because patients can have >1 reaction. Reactions also included 790 other reactions and 50 unknown reactions.

i Nonsevere cutaneous reactions to cephalosporins included rash (n = 109), hives (n = 45), itching (n = 19), and flushing (n = 2).

j Severe IgE reactions to cephalosporins included anaphylaxis (n = 18), angioedema (n = 9), swelling (n = 8), shortness of breath (n = 4), hypotension (n = 2), arrhythmia (n = 2), and wheezing (n = 1).

### Study design overview

We identified all PHS β-lactam antibiotic test doses performed from April 2016 through December 2017. Although β-lactam test doses were not performed at community hospital sites prior to April 2016, test doses at academic sites prior to guideline adoption occurred exclusively at the direction of an allergist. The β-lactam antibiotic test doses reviewed included those performed with and without preceding PST; PST was available at 3 sites by an allergy/immunology consultation. All patients receiving 1 or more test-dose challenge had their EHR reviewed by PHS house staff, with data entry and maintenance supported by research electronic data capture (RedCap) hosted by PHS.^17^

### Data definitions and outcomes

The EHR-abstracted data included characteristics of the test dose (ie, drug, hospital, ordering clinician, patient care service, allergy/immunology consultation use, PST use, test-dose timing, and length of stay) and patient characteristics (ie, demographics and allergy history). Historical penicillin and cephalosporin reactions were recorded. Itching, flushing, rash, and hives were considered nonsevere cutaneous reactions; bronchospasm, shortness of breath, wheezing, anaphylaxis, angioedema, swelling, syncope, arrhythmia, hypotension, dizziness, and positive skin testing were considered severe IgE histories. Other EHR drug allergies were recorded.

The primary outcome was a hypersensitivity reaction (HSR) resulting from a β-lactam antibiotic test dose. PHS house staff reviewers recorded reaction details including timing or onset, test-dose step, symptoms and presentation, treatment, and clinical context for all possible reactions. Allergy specialist coinvestigators (K.G.B., P.G.W., J.T.H., and A.R.W.) determined whether the signs and/or symptoms were consistent with an HSR. All reactions not consistent with HSRs were considered adverse drug reactions (ADRs). For all HSRs, allergy specialists determined whether objective findings were present and whether the pathways were followed correctly. Confirmed HSRs were grouped as follows. Nonsevere cutaneous reactions included itching, flushing, rash, tingling, and urticaria; severe IgE reactions included angioedema, swelling, bronchospasm, wheezing, hypotension, and anaphylaxis; and severe delayed immunologic reactions included organ-specific reactions and severe cutaneous adverse reactions. We assessed HSRs overall by drug class, and we separately considered direct challenges (ie, challenges performed without prior PST).

Modification of the EHR allergy record after a test dose was determined by assessing whether the allergy module had a relevant allergy entry added, deleted, and/or specified (ie, additional detail was included).

### Statistical analysis

We used descriptive statistics such as numbers with frequencies and medians with interquartile ranges. Univariable comparisons were made using χ^2^ and Kruskal-Wallis tests. Confidence intervals (CIs) were calculated using exact (ie, Clopper Pearson) methods. The HSR and ADR predictors were identified using multivariable logistic regression models, and we reported adjusted odds ratios (aORs) with 95% confidence intervals (CIs). The selection of variables to include in multivariable models involved *a priori* knowledge of variable association with outcome. Statistical analyses were conducted using SAS version 9.4 software (SAS Institute, Cary, NC).

## Results

### Test-dose characteristics

From April 2016 through December 2017, 1,046 test doses to β-lactams were administered to 942 patients across 5 PHS hospitals (Table 1). Test-dose procedures were performed to penicillins (n = 148), cephalosporins (n = 809), and carbapenems (n = 89). Test doses were performed largely at academic sites (83%) by house staff (59%). The most common service performing test-dose procedures was internal medicine (45%).

Allergy/Immunology staff were consulted for 96 (9%) test-dose challenges administered, more often for penicillin test doses (19%) than for carbapenem test doses (16%) or cephalosporins (7%; *P* < .001). A PST prior to the test dose was performed for 38 patients (4%), most commonly prior to penicillin test doses (13%), compared to cephalosporins test doses (2%) and carbapenem test doses (0%; *P* < .001).

Patients were in the hospital a median of 2 days prior to their test dose (interquartile range [IQR], 1–4 days); patients received penicillin test doses later in the hospitalization (3 days; IQR, 1–7 days) than patients who received cephalosporin (2 days; IQR, 1–4 days) or carbapenem test doses (2 days; IQR, 1–6 days; *P* = .003). The overall median length of stay was 10 days (IQR, 5–19 days) for patients receiving test doses.

### Patient characteristics

Patients receiving test doses were mostly female (65%) with a median age of 64 years (IQR, 51–75). Patients receiving test doses had penicillin allergy histories (96%); 29% had cephalosporin allergy histories. Penicillin allergy histories included nonsevere cutaneous reactions (48%) and severe IgE-mediated reactions (20%). Cephalosporin allergy histories included nonsevere cutaneous reactions (17%) and severe IgE-mediated reactions (5%).

### Adverse and hypersensitivity reactions

We identified 78 ADRs (7.5%; 95% CI, 5.9%–9.2%), of which 40 (3.8%; 95% CI, 2.8%–5.2%) were HSRs and 38 (3.6%; 95% CI, 2.6%–5.0%) were non-HSRs (eg, somatic symptoms, intolerances, or toxicities).

Most HSRs occurred after 24 hours from the initial test dose (n = 16, 40%), but 14 (35%) occurred during the 1-hour test-dose observation period (Table 2). HSRs were nonsevere cutaneous reactions (n = 25, 63%). Symptoms suggestive of severe IgE-mediated reactions (n = 10, 25%) and severe delayed HSRs (n = 3, 8%) were also observed. Most HSRs required no treatment (n = 23, 58%). Treatments included antihistamines (n = 16, 40%), parenteral corticosteroids (n = 3, 8%), and epinephrine (n = 3, 8%). Objective findings had been recorded for most HSRs (n = 34, 85%). The pathway was interpreted correctly in most cases (n = 34, 85%); however, the pathway was not followed correctly for 1 of the 3 patients treated with epinephrine.

**Table 2.** Hypersensitivity Reactions Resulting From β-Lactam Test-Dose Challenge Procedures

Variable	Hypersensitivity Reactions (n = 40)
**Reaction timing, h**	
≤1 h	14 (35)
> 1 h to <4 h	4 (10)
≥ 4 h to <24 h	2 (5)
> 24 h	16 (40)
Unknown	4 (10)
**Symptoms or presentation**	
Nonsevere cutaneous reactions^a^	25 (63)
Severe IgE reactions^b^	10 (25)
Severe delayed reaction^c^	3 (8)
**Reaction treatment**	
None	23 (58)
Antihistamines	16 (40)
Parenteral corticosteroids	3 (8)
IM epinephrine	3 (8)^d^
Albuterol	1 (3)
Unknown	13 (33)
Objective findings	34 (85)
Pathway followed and correctly implemented	34 (85)^e^

Note. IM, intramuscular; PST, penicillin skin test.

a Includes rash (n = 19), itching (n = 6), hives (n = 2), tingling (n = 1). Numbers do not sum because patients can have >1 reaction.

b Includes bronchospasm/wheezing (n = 5), angioedema/swelling (n = 4), hypotension/dizziness (n = 3), anaphylaxis (n = 1). Numbers do not sum because patients can have >1 reaction.

c Includes acute interstitial nephritis (n = 1), severe cutaneous adverse reaction (n = 1), and acute generalized exanthematous pustulosis (n = 1)

d All 3 patients whose HSR required IM epinephrine treatment had cephalosporin test-dose challenges. The first patient had a history of urticaria and angioedema to penicillin and developed throat tightness, diffuse pruritus, abdominal pain, and wheezing during the cefepime full dose; IM epinephrine, hydroxyzine, and albuterol led to resolution. The second patient had a history of ampicillin anaphylaxis and received ceftriaxone by test dose and full dose uneventfully, but developed throat tightness when broadened to cefepime for *Pseudomonas* spp coverage. Symptoms resolved with IM epinephrine, parenteral steroids, and antihistamines. The third patient had a history of penicillin anaphylaxis and was administered cefoxitin by test dose without prior PST. The patient experienced blurry vision, throat closing, and diffuse pruritus; symptoms resolved with IM epinephrine and diphenhydramine.

e Pathway was not followed because: patient was too sick/deemed inappropriate candidate for test dose (n = 3), patient had active allergy symptoms (n = 2), or the algorithm was not correctly interpreted (n = 1).

An allergy to cephalosporin antibiotics (adjusted odds ratio [aOR], 2.96; 95% CI, 1.34–6.58) was associated with increased odds of an HSR (Supplemental Table 2 online). Female sex (aOR, 1.86; 95% CI, 1.11–3.13), allergy to cephalosporin antibiotics (aOR, 2.49; 95% CI, 1.37– 3.13), and allergy consultation (aOR, 2.42; 95% CI, 1.30–4.51) were associated with significantly increased odds of an ADR.

### Hypersensitivity reactions to direct test-dose challenges

**Fig. 1.**
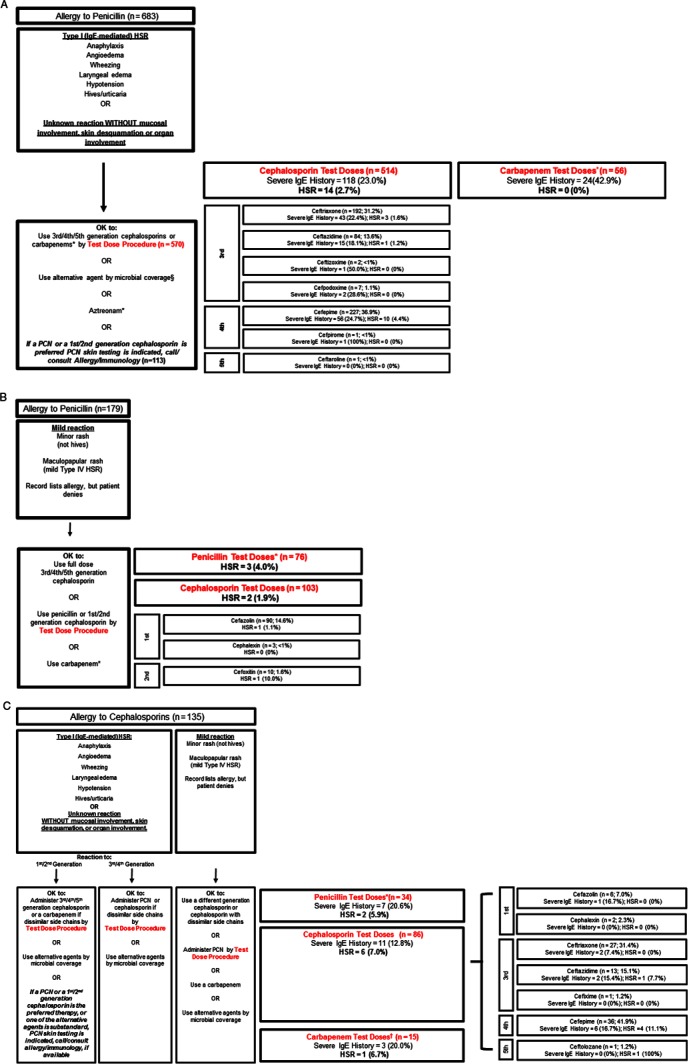
Reactions from direct β-lactam antibiotic challenges. These figures provide insight into β-lactam hypersensitivity reactions (HSRs) and real-world potential cross-reactivity in acute care patients with well-characterized historical reactions. Patients exclude patients with both penicillin and cephalosporin allergy histories and those who received penicillin skin testing (PST) prior to their challenge. (A) Patients with penicillin allergy histories who received β-lactam test doses following the penicillin hypersensitivity pathway through the type 1 (IgE-mediated) HSR pathway (n = 683). *Meropenem (n = 46), imipenem (n = 6), ertapenem (n = 4). (B) Patients with penicillin allergy histories who received β-lactam test doses following the penicillin hypersensitivity pathway through the mild HSR pathway (n = 179). *Piperacillin/tazobactam (n = 26), ampicillin/sulbactam (n = 21), ampicillin (n = 13), amoxicillin (n = 6), penicillin G (n = 5), nafcillin (n = 4), amoxicillin/clavulanic acid (n = 1). (C) Patients with cephalosporin allergy histories who received β-lactam test doses following the cephalosporin hypersensitivity pathway (n = 135). *Piperacillin/tazobactam (n = 18), ampicillin/sulbactam (n = 8), ampicillin (n = 5), nafcillin (n = 2), penicillin G (n = 1). ^†^Meropenem (n = 11), ertapenem (n = 3), imipenem (n = 1). Note. Ig, immunoglobulin; PCN, penicillin.

Overall, 570 penicillin allergy patients who had IgE histories or unknown histories directly challenged with cephalosporins (third, fourth, or fifth generation) or carbapenems [Fig. 1(A)]. Of the 514 patients directly challenged with third-, fourth-, or fifth-generation cephalosporins, 14 (2.7%) had HSRs (95% CI, 1.5%–4.5%). The highest HSR rate was to cefepime (4.4%; 95% CI, 2.1%–8.0%). Of 56 patients direct challenged to carbapenems, none had HSRs.

We identified 179 patients with mild penicillin reaction histories who received direct test-dose challenges. Of 76 patients direct challenged to penicillin, 3 had HSRs (4.0%; 95% CI, 0.8%–11.1%). Of 103 patients directly challenged with cephalosporins (first or second generation), 2 patients exhibited HSRs (1.9%; 95% CI, 0.2%–6.8%) [Fig. 1(B)].

There were 135 patients with cephalosporin allergy histories directly challenged with β-lactams: 34 with penicillins (2 HSRs, 5.9%; 95% CI, 0.7%–19.7%), 86 with cephalosporins (6 HSRs, 7.0%; 95% CI, 2.6%–14.6%), and 15 with carbapenems (1 HSR, 6.7%; 95% CI 0.2%–32.0% [Fig. 1(C)].

### Updating the electronic health record allergy module

Overall, EHR allergy sections of 474 of 1,046 patients (45%) were updated after the β-lactam antibiotic test-dose challenge. Among the updated cases, 37 (8%) had an allergy added, 75 (16%) had an allergy deleted, and 390 (82%) had an allergy specified (records could have >1 action). The PHS community hospitals updated the EHR more frequently after test doses than did PHS academic hospitals (54% vs 43%; *P* = .009). Of patients who had had an allergy/immunology consultation (n = 96), the EHR was updated for 59 (61%).

## Discussion

We implemented a healthcare system-wide guideline to standardize the approach to inpatients with β-lactam allergy histories as an antibiotic stewardship tool.^16^ We report the outcomes of 1,046 β-lactam antibiotic test-dose challenges performed by 5 diverse acute-care hospitals within a single healthcare system, largely without preceding PST (96%). Test doses were predominantly to cephalosporin antibiotics. The ADR rate was 7.5% and the HSR rate was 3.8%. Most HSRs occurred after the full-dose step and required no treatment beyond drug discontinuation. Among 10 HSRs consistent with severe IgE-mediated HSRs, 3 were treated with intramuscular epinephrine. Although a cephalosporin allergy history conferred a 3-fold increased HSR risk, HSRs to cephalosporins were infrequent in patients with specific penicillin allergy histories. Allergy records after test doses were not routinely updated.

Hospitalized patients with documented β-lactam allergies experience inferior outcomes, including treatment failures, adverse events, resistant organisms, and healthcare-associated infections.^3^^,^^4^^,^^6^^–^^8^ To address this problem, hospitals implemented structured allergy histories, PST, and/or comprehensive guidelines.^11^^,^^12^^,^^18^^–^^20^ Because PST can pose operational challenges,^12^ effective skin testing interventions often select inpatients for PST evaluation based on “need,” such as patients on broad-spectrum or nonpreferred antibiotics,^21^^–^^23^ or patients referred through an infectious diseases consultation.^18^^,^^24^ When more inclusive inpatient PST studies were attempted, only 20%–33% of eligible inpatients underwent testing.^11^^–^^13^ Our guideline uses PST only when indicated, given both the allergy history and the desired therapeutic antibiotic, and when institutionally available. This guideline applies to all adults, children, and pregnant women in all care units (eg, emergency departments, medical wards, and intensive care units). Previously, this guideline increased β-lactam use by 80%,^12^ and in this study of >1,000 test doses, it was safe and feasible in a large, diverse healthcare system. Notably, hospitals without access to inpatient allergy/immunology consultation or PST were included, and 1 such site (Newton Wellesley Hospital, NWH), contributed almost 10% of the test doses analyzed. NWH’s high test-dose volume may have been facilitated by shared MGH house staff, who had been implementing the guideline at MGH beginning in 2013, 3 years prior to other sites. Although NWH and North Shore Medical Center had consistent clinical champions since program implementation, clinical champion turnover at the Brigham and Women’s Faulkner Hospital may have impacted their test-dose volume. Nevertheless, each site achieved successful implementation regardless of access to an allergist. Given that most US hospitals lack immediate allergist or PST access, our approach may be widely feasible. Indeed, some US hospitals have already adopted this approach, and pathways were incorporated into educational materials.^19^^,^^25^^,^^26^^,^^27^

In this study, 7.5% of test-dose challenges resulted in an ADR. Notably, a nocebo effect (ie, noxious symptoms from a placebo drug) can occur in patients with prior drug reactions.^28^^,^^29^ A recent US study reported that 10% of patients who thought they were challenged to amoxicillin in an outpatient allergy practice “reacted” to the placebo.^30^ ADR risk factors included female sex, patients with cephalosporin allergy histories (also a significant HSR risk factor), and allergy/immunology consultation. Female sex was previously associated with higher rates of reported drug allergies/intolerances.^31^^,^^32^ Allergy/Immunology consultation was associated with a higher ADR risk by design: consultations were indicated after positive challenges in locations with allergy/immunology access. The overall ADR frequency is important to consider; all patient-reported symptoms require assessment, and although reassurance may be possible for patients with only subjective symptoms, ADRs after test-dose procedures often result in drug discontinuation.

The HSR rate observed in this study was 3.8%, which is similar to our prior study at MGH (3.9%) and comparable to the penicillin HSR rate observed previously by allergy specialists (1.5%–2.6%).^30^^,^^33^ Furthermore, all drug administration carries a comparable risk; hospitalized patients with infections have a baseline incidence of antibiotic allergy between 0.5% and 5.0%.^34^ The HSRs in this study were determined by allergist review and were largely supported by objective data. Although ∼25% of HSRs had signs or symptoms suggestive of a severe IgE-mediated HSR, most HSRs required no antiallergy treatment, and only 3 HSR patients were treated with epinephrine. More than one-third (35%) of HSRs were triggered by the test dose, which may have led to those 14 patients having less severe HSRs.

This study provides insight into “real world” β-lactam cross reactivity in patients with specified IgE penicillin allergy histories, including patients with severe IgE histories. Prior observational studies of cephalosporins administered to penicillin allergy patients largely excluded patients with higher-risk penicillin allergy histories because they were selected out.^5^ Patients with severe IgE histories have an increased risk of true allergy and β-lactam cross reactivity.^35^^,^^36^ Although the later (third-, fourth-, or fifth-) generation cephalosporins overall had a low 2.6% HSR rate in patients with IgE penicillin allergy histories, the cefepime HSR rate was 4.4% with a 95% CI of up to 8.0%. It remains unclear whether later-generation cephalosporins need to be initiated with a test-dose challenge (many clinicians initiate these cephalosporins in penicillin allergy histories with a full dose). Of 56 carbapenem test-doses administered to patients with IgE penicillin allergy histories (including almost half with severe IgE histories), there were no HSRs, which prompts us to consider modification of the penicillin hypersensitivity pathway to indicate that carbapenems be administered by a full dose. For mild penicillin allergy histories, full-dose challenges for first- or second-generation cephalosporins are a safe modification that would facilitate the use of any cephalosporin for any patient with mild penicillin allergy histories. This change could benefit acute-care obstetric and perioperative patients where cefazolin or cefoxitin are indicated.^3^^,^^37^

Allergy to cephalosporin antibiotics was associated with a significant 3-fold increased odds of HSR. Documented cephalosporin allergies may more often be true hypersensitivities that occurred more recently compared to documented penicillin allergies (often “unknown” and/or remote). The cephalosporin hypersensitivity pathway may not be as accurate as the penicillin hypersensitivity pathway in this US acute-care populaton.^38^^,^^39^ Although a notable signal, because only 135 patients with cephalosporin allergy histories received direct cephalosporin test doses to date, more data gathering on this approach is needed prior to considering pathway modifications.

Drug allergy documentation is important for quality and safety. However, EHR allergy documentation is often incomplete and inaccurate.^40^ Inpatient β-lactam allergy interventions require taking an allergy history that should then be recorded in the EHR. Furthermore, results of skin tests and drug challenges should be specified in the EHR to ensure communication between providers and settings.^41^^,^^42^ We identified that allergy documentation was changed less than half of the time after test doses were performed. Incomplete documentation compromises the effectiveness of any allergy intervention as an antibiotic stewardship tool; β-lactams may be unnecessarily avoided or an allergy procedure might be repeated unnecessarily. Targeted alerting to the test dose prescriber may improve allergy documentation after test doses.

Although the guideline can recommend β-lactam avoidance or administration of a full-dose β-lactam, we only reviewed β-lactams initiated by a test dose in this study. All data were abstracted and analyzed retrospectively, which can result in misclassification; however, HSR determination was rigorously determined by allergist coinvestigators. While evaluating HSRs occurring from direct challenges in patients with different allergy histories provides insight into beta-lactam cross-reactivity, patients in this study had an unknown true allergy status. Although we evaluated HSRs occurring from direct challenges in patients with different allergy histories provides insight into β-lactam cross reactivity, we were unable to obtain the true allergy statuses of patients included in this study. Finally, we report on a multisite intervention in which all hospitals are part of a single geographically localized large US healthcare system. However, non-PHS hospitals have also adopted this guideline.^19^

An electronic guideline with penicillin and cephalosporin hypersensitivity pathways encouraging β-lactam test-dose challenges was implemented in 5 hospitals with different resources using 1 EHR as an antibiotic stewardship tool. The ADR and HSR rates were low, and not higher than expected given a morbid inpatient population with prior reported drug allergies. Certain test-dose challenges may be omitted given their observed safety, and caution is prudent with cephalosporin-allergy challenged inpatients. Additional efforts to improve allergy documentation are important to the success of β-lactam allergy interventions.

## Supplementary material

For supplementary material accompanying this paper visit https://doi.org/10.1017/ice.2019.50. click here to view supplementary material
