# Supplementary material for: Outcomes from an inpatient beta-lactam allergy guideline across a large US health system
Source: Infect Control Hosp Epidemiol. 2019 May;40(5):528–35. doi: 10.1017/ice.2019.50 (PMC6536839; doi:10.1017/ice.2019.50)
Supplement: Supplementary file 1 [file S0899823X19000503supp001.docx]

Outcomes from an Inpatient Beta-Lactam Allergy Guideline Across a Large US Health System

Kimberly G. Blumenthal, MD, MSc^1,2,3^

Yu Li, MS^4^

Joyce T. Hsu, MD^3,5^

Anna R. Wolfson, MD^1,3^

David N. Berkowitz, PharmD^6^

Victoria A. Carballo, MPH^7^

Jesse M. Schwartz, MD, MSc, MPH^8^

Kathleen A. Marquis, PhD, PharmD^4,9^

Ramy Elshaboury, PharmD, BCPS-AQ ID^10^

Ronak G. Gandhi, PharmD, BCPS^10^

Barbara B. Lambl, MD, MPH^11^

Monique M. Freeley, RPh^12^

Alana Gruszecki, PharmD, RPh, BCPS^13^

Paige G. Wickner, MD, MPH^3,5*^

Erica S. Shenoy, MD, PhD^3,14,15*^

^1^Division of Rheumatology, Allergy, and Immunology, Department of Medicine, Massachusetts General Hospital, Boston, MA

^2^ The Mongan Institute, Massachusetts General Hospital, Boston, MA

^3^Harvard Medical School, Boston, MA

^4^ University of Pittsburgh School of Medicine, Pittsburgh, PA

^5^ Division of Rheumatology, Allergy and Immunology, Department of Medicine, Brigham and Women’s Hospital, Boston, MA

^6^ Department of Pharmacy, Newton-Wellesley Hospital, Newton, MA

^7^ Partners HealthCare System, Quality, Safety, and Value, Boston, MA

^8^ Division of Allergy and Clinical Immunology, Jewish General Hospital, McGill University, Montreal, Quebec, CA

^9^ Department of Pharmacy, Brigham and Women’s Hospital, Boston, MA

^10^ Department of Pharmacy, Massachusetts General Hospital, Boston, MA

^11^ Division of Infectious Diseases, Department of Medicine, North Shore Medical Center, Salem, MA

^12^ Pharmacy Department, North Shore Medical Center, Salem, MA

^13^ Pharmacy Department, Brigham and Women’s Faulkner Hospital, Boston, MA

^14^ Division of Infectious Diseases, Department of Medicine, Massachusetts General Hospital, Boston, MA

^15^ Infection Control Unit, Massachusetts General Hospital, Boston, MA

^*^ These authors contributed equally

**Corresponding Author**

Kimberly G. Blumenthal, MD, MSc, Division of Rheumatology, Allergy and Immunology, The Mongan Institute, Massachusetts General Hospital 100 Cambridge Street, 16^th^ Floor, Boston, MA 02114**,** p-(617) 726-3850, f-(617) 724-7441**,** kblumenthal@mgh.harvard.edu

**Abbreviated Title:** Beta-Lactam Allergy Guideline Outcomes

**Contents:**

Supplemental material: Tables (2)

**Supplemental Table 1.** Hospital characteristics

|  | **MGH** | **BWH** | **NWH** | **NSMC*** | **BWF** |  |
| --- | --- | --- | --- | --- | --- | --- |
| **Hospital Type** | Academic | Academic | Community^‡^ | Community | Community^†^ |  |
| **Massachusetts Location** | Boston | Boston | Newton | Salem/Lynn | Boston |  |
| **Number of Beds**^§^ | 1,035 | 793 | 265 | 396 | 162 |  |
| **Number of Annual Admissions**^║^ | 49,688 | 42,192 | 15,409 | 17,377 | 10,033 |  |
| **Clinical champion** | Allergy physician | Allergy physician | Pharmacist | Infectious Diseases  physician | Hospitalist  Physician** |  |
| **Antibiotic Stewardship Program Members** | Physicians, Pharmacists, Infection Control, Clinical Microbiology | Physicians, Pharmacists, Infection Control, Clinical Microbiology | Physicians, Pharmacists, Infection Control,  Clinical Microbiology,  Nursing | Physicians, Pharmacists | Physicians, Pharmacists |  |
| **Allergy/Immunology Consultation Available** | Yes | Yes | No | Yes | No |  |
| **Inpatient penicillin skin testing available** | Yes | Yes | No | Yes | No |  |
| ^*^ NSMC is comprised of Salem Hospital and Union Hospital  ^‡^ Housestaff from MGH  ^†^ Housestaff from BWH  ^§^ Licensed beds of 9/30/17  ^║^ October 1, 2016 through September 30, 2017  **Clinical champion during study period was not consistent; a new hospitalist joined our group 07/2017  *Abbreviations:* MGH, Massachusetts General Hospital; BWH, Brigham and Women’s Hospital; NWH, Newton Wellesley Hospital; NSMC, North Shore Medical Center; BWF, Brigham and Women’s Faulkner Hospital; ICU, Intensive Care Unit | | | | | | |

**Supplemental Table 2.** Patient and hospital predictors of hypersensitivity reactions and adverse drug reactions.

| **Hypersensitivity Reactions** | **Odds ratio [95% CI]*** | **p-value** |
| --- | --- | --- |
| Female | 1.33 [0.68, 2.60] | 0.41 |
| Age | 0.99 [0.98, 1.01] | 0.47 |
| Allergy to penicillin | 1.08 [0.43, 2.71] | 0.87 |
| Allergy to cephalosporin | 2.96 [1.34, 6.58] | 0.008 |
| Historical reaction severe IgE | 1.59 [0.78, 3.25] | 0.20 |
| Allergy consultation | 1.83 [0.77, 4.35] | 0.17 |
| Ordering provider not a medical doctor | 1.12 [0.55, 2.27] | 0.76 |
| Community hospital | 0.61 [0.21, 1.78] | 0.36 |
| **Adverse Drug Reactions** | **Odds ratio [95% CI]*** | **p-value** |
| Female | 1.86 [1.11, 3.13] | 0.02 |
| Age | 1.00 [0.99, 1.01] | 0.73 |
| Allergy to penicillin | 1.18 [0.58, 2.42] | 0.65 |
| Allergy to cephalosporin | 2.49 [1.37, 4.51] | 0.003 |
| Historical reaction severe IgE | 1.27 [0.74, 2.19] | 0.38 |
| Allergy consultation | 2.42 [1.30, 4.51] | 0.005 |
| Ordering provider not a medical doctor | 1.00 [0.53, 1.56] | 0.74 |
| Community hospital | 0.59 [0.27, 1.29] | 0.19 |
| *Multivariable logistic regression model results | | |
